# Integrated aerobic exercise with LDE-docetaxel treatment: a novel approach to combat prostate cancer progression

**DOI:** 10.1038/s41598-024-60138-y

**Published:** 2024-04-26

**Authors:** Allice Santos Cruz Veras, Victor Rogério Garcia Batista, Rafael Ribeiro Correia, Maria Eduarda de Almeida Tavares, Rafael Jesus Gonçalves Rubira, Elaine Rufo Tavares, Inês Cristina Giometti, Raul Cavalcante Maranhão, Giovana Rampazzo Teixeira

**Affiliations:** 1 Multicenter Graduate Program in Physiological Sciences, SBFis, São Paulo State University (UNESP), Presidente Prudente, SP Brazil; 2 Department of Physical Education, São Paulo State University (UNESP), School of Technology and Sciences, Presidente Prudente, Street Roberto Simonsen, 305, Presidente Prudente, SP 19060-900 Brazil; 3 Department of Physics, School of Technology and Applied Sciences, São Paulo State University, Presidente Prudente, SP Brazil; 4 Postgraduate Animal Science Program, University of Western São Paulo (UNOESTE), Presidente Prudente, Brazil; 5 Lipid Metabolism Laboratory, Heart Institute (INCOR), Medical School Hospital, University of São Paulo, (USP), Presidente Prudente, SP Brazil

**Keywords:** Nanoemulsion, Raman spectroscopy, LDE, Endurance physical training, Taxanes, Cell biology, Urology

## Abstract

The variability in response to conventional prostate cancer (PC) therapies, coupled with the emergent issue of drug resistance, underscores the critical need for innovative treatment strategies. Aerobic physical exercise reduced incidence of several cancers, but the mechanism underlying these effects associated the nanoemulsion not fully understood. The application of a lipid nanoemulsion (LDE) delivery system for docetaxel (DTX), showing marked enhancement in therapeutic efficacy when combined with aerobic physical exercise. This novel intervention potentiates the antitumor activity of LDE-delivered DTX by augmenting nanoparticle internalization and inducing cell cycle arrest. Our findings reveal that this synergistic treatment not only significantly reduces prostate weight and mitigates adenocarcinoma proliferation but also attenuates anti-apoptotic BCL-2 protein expression. Concurrently, it elevates pro-apoptotic proteins and diminishes inflammatory markers. Metabolic profiling of the combined therapy group disclosed additional benefits, such as reduced lipid and plasma glucose levels. Collectively, our data illuminate the profound impact of integrating LDE-mediated DTX delivery with structured physical exercise, which together spearhead a dual-front assault on PC. This multimodal approach heralds a new paradigm in PC management, accentuating the promise of combined pharmacological and non-pharmacological interventions to elevate tumor suppressor protein activity and refine patient outcomes.

## Introduction

Prostate cancer (PC) is the second most prevalent cancer in males accounting for 20% of all newly diagnosed cancers, affecting more than one million men each year^1^. PC is a hormone-dependent tumor that in cases that relapse after radical prostatectomy or radiotherapy are submitted to androgen deprivation therapy (ADT). Unfortunately, PC develop resistance to ADT and chemotherapy is indicated, and combined platines and taxanes are used in first line schemes^2^. In this setting, the control of the disease is challenging, and new treatment approaches are welcome to improve progression free period and longer overall survival of those patients.

Groundbreaking work by Maranhão et al. has established a new frontier in targeted cancer therapy by harnessing lipidic nanoparticles mimicking low-density lipoprotein (LDL) structures. These nanoparticles exhibit an affinity for LDL receptors, achieving preferential localization and heightened drug delivery within neoplastic cells and tissues^3^. Exploiting the elevated mitotic activity and cholesterol demand of cancer cells, these LDL-like nanoemulsions—devoid of protein yet capable of acquiring apolipoproteins such as apo E from plasma—leverage the endocytic pathway mediated by LDL receptors for cellular uptake^3,4^. Termed LDEs to denote their LDL mimicry and apo E binding, these nanoemulsions have shown substantial promise. When paired with taxanes like paclitaxel and docetaxel, LDEs significantly mitigate toxicity and enhance pharmacological efficacy^3^.

Clinical trials involving LDE-paclitaxel have yielded encouraging results in terminal-stage patients suffering from ovarian, breast, lung, and prostate cancers with bone metastasis, demonstrating negligible toxicity and suggestive therapeutic advantages. Additionally, docetaxel loaded LDEs have been explored in rabbit models of atherosclerosis induced by cholesterol diets, where they not only attenuated atherosclerotic lesions but also exhibited pronounced anti-inflammatory effects associated to LDE was tested only in rabbits with atherosclerosis induced by cholesterol feeding^5^. This innovative approach not only paves the way for more effective chemotherapeutic regimens but also underscores the potential of LDE-docetaxel in the broader context of inflammatory pathologies.

Physical exercise is well-documented to confer a multitude of benefits across various physiological domains, including metabolic regulation, cardiovascular health, and cellular plasticity^6–8^. Within the realm of oncology, a pivotal mechanism underlying the beneficial effects of exercise is its role in apoptotic modulation via the p53 pathway in prostate cells, suggesting a potential oncogenic attenuation^9–11^. Concurrently, exercise-induced lipid modulation has been implicated in the regulation of serum cholesterol and glucose concentrations, thereby potentially curtailing the metabolic support of neoplastic cells^12,13^.

Our previous research has established that aerobic physical exercise can attenuate the progression of prostate cancer^11,14^. The present inquiry seeks to elucidate the synergistic effects of physical activity when coupled with conventional prostate cancer treatments. In the context of lipid metabolism, it has been demonstrated that athletes exhibit a fivefold increase in the systemic clearance of LDE compared to sedentary individuals^15^. Further corroborating this, experimental models utilizing LDL receptor knockout mice subjected to exercise regimens have revealed an overexpression of LDL receptors, which may elucidate the enhanced clearance of LDE^16^. These findings advocate for a deeper investigation into the adjunctive role of physical exercise in the therapeutic management of prostate cancer.

We investigated the anti-cancer effectiveness of LDE-docetaxel, a lipid nanoemulsion that may enhance tumor targeting via LDL receptors and modulate apoptotic proteins. Our study delved into the interaction between nanoparticles and cancer cells, examining impacts on cell cycle and apoptosis, as well as tumor biomarkers in the blood. We considered the possibility that physical exercise might increase the uptake of LDE-docetaxel by cancer cells, thereby boosting its anti-cancer activity. Using a prostate cancer model, we found that LDE-docetaxel regulates oncogenic protein levels. Our data indicate that physical exercise amplifies the effects of LDE-docetaxel, leading to a reduction in prostatic adenocarcinoma. This study primarily aims to assess the anti-cancer potential of LDE-docetaxel in an animal model of prostate cancer and determine whether physical exercise can enhance its efficacy. Our findings suggest that combining physical training with LDL receptor-targeted chemotherapy may represent a novel and effective approach to prostate cancer treatment.

## Results

### Body weight, feeding efficiency, tumoral volume and incremental load test

**Figure 1:**
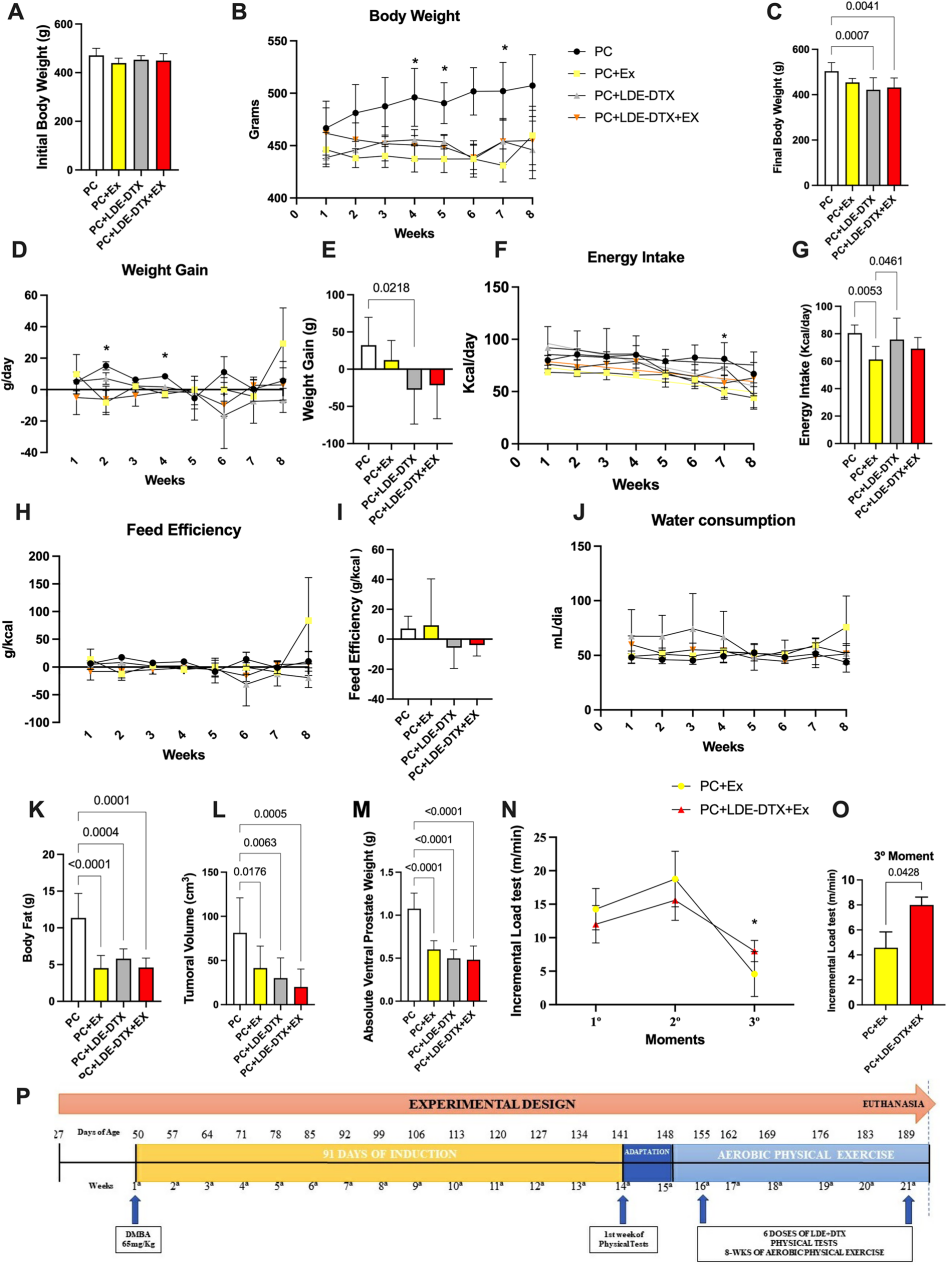
(**A**) Initial body mass (g) (**B**) Animals body weight referring only to the 8 weeks of physical exercise period (g); (**C**) Final body mass (g); (**D**) Weight gain referring only to the 8 weeks of physical exercise period (g/day); (**E**) Total weight gain along the experimental protocol (g/day); (**F**) Energy intake referring only to the 8 weeks of physical exercise period (Kcal/day); (**G**) Total energy intake (Kcal/day); (**H**) Feed efficiency referring only to the 8 weeks of physical exercise period (g/kcal); (**I**) Total feed efficiency (g/Kcal); (**J**) Water consumption intake referring only to the 8 weeks of physical exercise period (mL/day); (**K**) Total fat of animals (g); (**L**) Tumoral volume (cm^3^); (**M**) final ventral prostate weight (g); (**N**) Incremental load test of 3 moments (in the beginning of experiment, at the middle of experiment, and in the final of experimental protocol); (**O**) Difference between PC-Ex and LDE-DTX + Ex in incremental load test in 3rd moment; (**P**) Experimental delimitation related to protocol during the 14 weeks.

To study the effects of treatments on nutrition and body composition we analyzed weekly food consumption and weight of animals from the beginning to the end of the protocol. The body weight of the animals at the beginning was similar among the six groups (Fig. 1A). Throughout the intervention period, in the fourth, fifth and seventh week, the animals in the PC + Ex group had lower body weight when compared to the PC group (Fig. 1B). However, after experimental treatment period the final body weight was significantly lower in the PC + LDE-DTX group (95% CI 30.89–132.3; *p* = 0.0007), e PC + LDE-DTX + Ex (95% CI 19.54–124.1; *p* = 0.0041) when compared to the PC group (Fig. 1C). Weight gain over the experimental treatment period varied with a significant difference at second week between the PC groups and the PC + Ex group (95% CI 2.689–43.92; *p* = 0.0360), in the fourth week between the PC + Ex groups (95% CI 7.798–14.88; *p* = 0.0018), LDE-DTX (95% CI 5.368–8.226; *p* = 0.0006) and PC + LDE-DTX + Ex (95% CI 5.585-13.85; *p* = 0.0045) when compared respectively with the PC group (Fig. 1D). Final weight gain was lower in the PC + LDE-DTX group compared to the PC group (95% CI 7.060–112.6; *p* = 0.0218; Fig. 1E).

To identify the relationship between weight loss and the growth of the tumor mass, we verified the energy intake and calculated the feed efficiency, as well as analyzed the volume of the tumor in the face of the treatments. Energy intake in the PC + Ex group had a significant reduction over in the seventh week compared to PC (p = 0.0354) and PC + LDE-DTX groups (*p* = 0.0043; Fig. 1F). The PC + Ex group showed energy intake compared to the PC group (95% CI 4.904–33.43; *p* = 0.0053) and PC + LDE-DTX group (95% CI − 28.72 to − 0.1936; *p* = 0.0461; Fig. 1G). Feed efficiency did not differ between groups (Fig. 1H,I). Water consumption did not differ between groups (Fig. 1J). Body fat reduced in PC + Ex (95% CI 3.484–10.17; *p* < 0.0001), PC + LDE-DTX (95% CI 2.353–8.750; *p* = 0.0004) and PC + LDE-DTX + Ex (95% CI 3.213–10.29; *p* = 0.0001) compared to PC group (Fig. 1K).

During the induction period and experimental protocol, there was a loss of approximately 12% of the total sample. Animals treated with PC + LDE-DTX and PC + LDE-DTX + Ex groups showed an effective reduction in tumor volume in experimental treatment period compared to the PC group (1.96-folds to PC + Ex, 2.79-folds to PC + LDE-DTX, and PC + LDE-DTX + Ex reduced about 4.04-folds, respectively; Fig. 1L). Most notably, the absolute prostate weight was lower in the PC + Ex, PC + LDE-DTX, and PC + LDE-DTX + Ex groups (78%, 116%, and 123% respectively) compared to the PC (Fig. 1M).

We performed incremental tests in 3 moments during the experimental treatment period. The incremental load test was similar between groups at baseline moment. Between moments 1 and 2, the animals PC + Ex and PC + LDE-DTX + Ex groups showed better performance (Fig. 1N). When analyzing moment 3, there was a reduction in physical capacity in both groups, where the PC + Ex group presented lower physical capacity when compared to the PC + LDE-DTX + Ex group (*p* = 0.0428, Fig. 1N,O). Therefore, aerobic physical exercise seems to have been more effective in maintaining physical capacity when associated with PC + LDE-DTX (Fig. 1N).

### Raman spectroscopy and biochemistry

**Figure 2:**
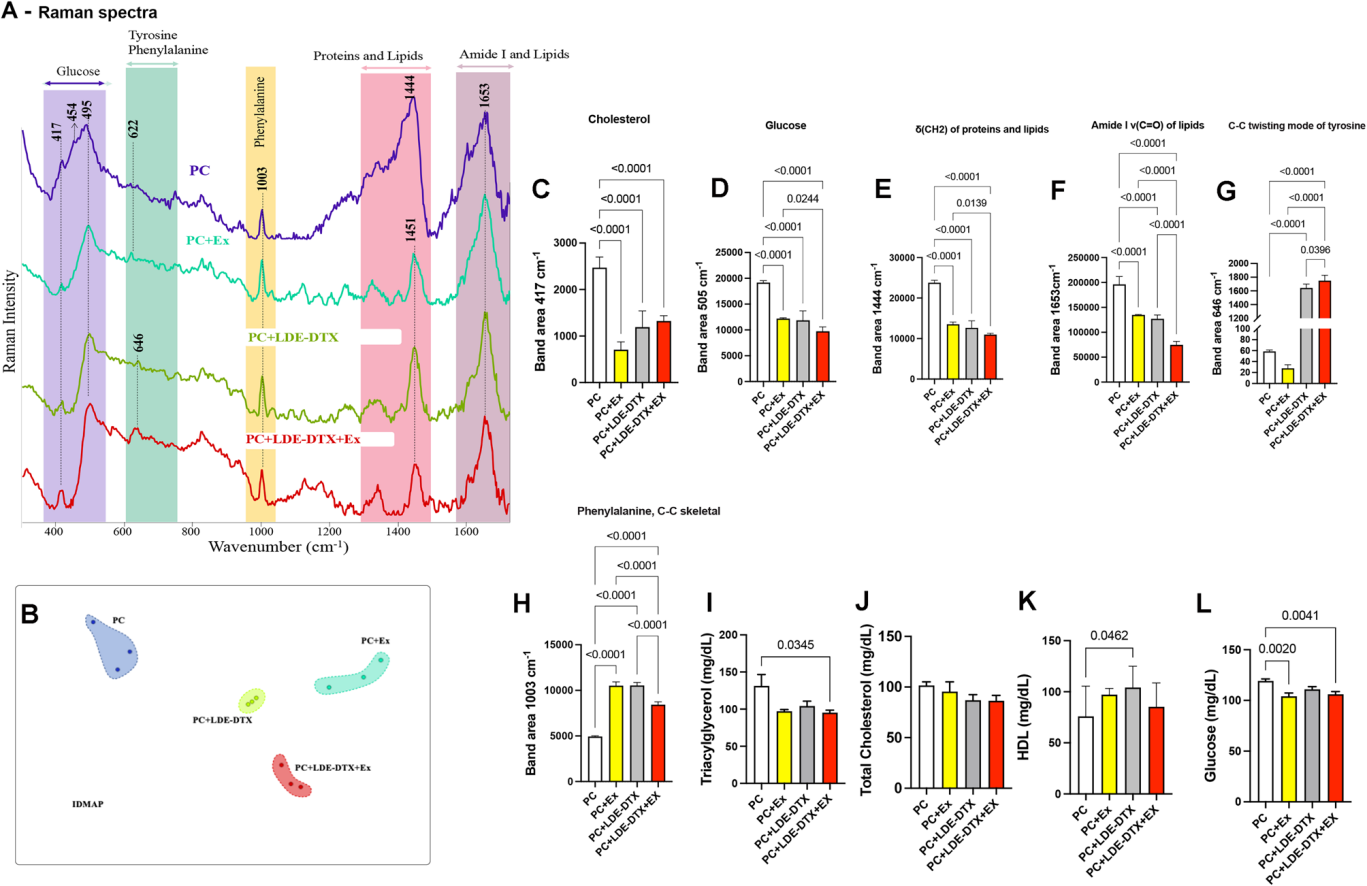
(**A**) Raman spectra in rats blood of pre-euthanasia collect; (**B**) Multidimensional IDMAP values of serum Raman spectra. (**C**) Cholesterol (cm^−1^); (**D**) Glucose (cm^−1^); (**E**) Protein and lipids findings (cm^−1^); (**F**) Amide I of lipids (cm^−1^); (**G**) Tyrosine (cm^−1^); (**H**) (cm^−1^); (**I**) Triacylglycerol findings (mg/dL); (**J**) Total cholesterol (mg/dL); (**K**) HDL (mg/dL); (**L**) Glucose (mg/dL). Raman spectra consists of several bands, each associated with a vibrational mode. The spectrum is unique to the material that enables you to identify it. In the graph’s, the y-axis represents the intensity of the scattered light, while the x-axis represents the energy (frequency) of found proteins.

Figure 2 shows the Raman spectra of blood serum. To evaluate spectral variance across the groups, we used principal IDMAP, as shown in Fig. 2B. Our results illustrate good clustering for different treatments (Fig. 2A) with greater variation in Raman spectra. The computational projection technique groups by similarities, but not only considers the difference between the Raman spectra but also the shape of the spectra shown in Fig. 2A.

All the assignments of the bands in low and high wavenumber are present in the Raman spectra. The intensity of the band 417 cm^−1^ cholesterol was lower in the PC + Ex groups (95% CI 1279–2255; *p* < 0.0001; Fig. 2A), PC + LDE-DTX (95% CI 792.2–1768; *p* < 0.0001; Fig. 2C) and PC + LDE-DTX + EX (95% CI 660.5–1636; *p* < 0.0001; Fig. 2C) compared to PC group (Fig. 2C). The sign of the intensity of the band 505 cm^−1^ that indicates the amount of glucose in blood was lower for the PC + LDE-DTX + Ex group when compared to the PC group (95% CI 7279–11,609; *p* < 0.0001; Fig. 2D) and PC + Ex group (95% CI 300.0–4631; *p* = 0.0244; Fig. 2D). Also, the PC group showed higher intensity values of the 505 cm^−1^ band when compared to the PC + Ex groups (95% CI 4813–9144; *p* < 0.0001; Fig. 2B) and PC + LDE-DTX (95% CI 5148–9479; *p* < 0.0001; Fig. 2D).

In general, the bands referring to lipids, protein, and amide I (1653, and 1444 cm^−1^) showed a higher Raman spectra area in the PC group in comparison the other groups (Fig. 2E,F). Raman spectra area in the band 1444–1451 cm^−1^ (protein and lipids) was 54% lower in the LDE-DTX + Ex group compared to the PC group (95% CI 10,739–14,838; *p* < 0.0001; Fig. 2E) and lower intensity compared to the PC + Ex group (95% CI 505.3–4604; *p* = 0.0139; Fig. 2E). For the 1653 cm^−1^ band (Amide I of lipids), the group treated with LDE-docetaxel and aerobic physical exercise showed lower area when compared to the PC group (95% CI 101,420–141,457; *p* < 0.0001; Fig. 2F), PC + Ex (95% CI 40,248–80,284; *p* < 0.0001; Fig. 2F) and LDE-DTX groups (95% CI 32,301–72,338; *p* < 0.0001; Fig. 2F). Another effect was the increase in the tyrosine and phenylalanine C–C skeletal (Fig. 2G and H), in the treated groups compared to the group PC (*p* < 0.0001).

Triacylglycerol levels were lower in PC + LDE-DTX + Ex group compared to the PC group (95% CI 2.048–69.81; *p* = 0.0271; Fig. 2I). The cholesterol levels have no significant difference between groups (Fig. 2J). In addition, HDL levels in serum from the PC + LDE-DTX group (95% CI 9.529–93.58; *p* = 0.002) were significantly higher when compared to PC group (Fig. 2K). A reduction in plasma glucose was observed in the PC + Ex (95% CI 4.895–25.33; *p* = 0.0020) and LDE-DTX + Ex (95% CI 3.595–22.71; *p* = 0.0041) compared to the PC group (Fig. 2L).

### LDE uptake

Table 1 shows LDE uptake. It was possible to verify that the animals submitted to physical exercise presented greater uptake of the LDE nanoemulsion compared to the group without physical exercise, with significant differences between the liver (*p* = 0.001) and prostate (*p* = 0.03).

**Table 1:** Tissue uptake of the 14C–CE by tissues in groups PC and PC-Exercise rats after injection of the LDE.

Tissues	Tissue uptake (%)		
	PC	PC-exercise	*p*-value
Liver	56.54 ± 3.9	63.47 ± 8.64	0.0186
Prostate	8.82 ± 0.57	12.47 ± 3.36	0.0304
Muscle	9.04 ± 3.76	7.60 ± 2.80	0.2613

Values are means ± SD of uptake of LDE cholesteryl ether in % of CPM total/g of tissue.

### Histopathology analysis

To understand the effect of the nanoemulsion LDE conjugated to DTX associated or not with physical exercise in prostate cancer, we verified the histopathology of the prostate of rats submitted to LDE-docetaxel treatment and to aerobic physical exercise. Table 2 showed histopathology analysis. The PC group had 58.71% of high-grade intraepithelial neoplasia (HGPIN) and a lower amount of low-grade intraepithelial neoplasia (LGPIN,) when compared to the other groups (Table 2). The PC + LDE + DTX and PC + LDE-DTX + Ex groups showed a reduction in the amount number of acinus with HGPIN and greater expression of LGPIN, reducing the degree of severity of the development of neoplasms (Table 2). There were no differences between groups in the amount number of acinus with metaplasia. The PC group had an average of 10% of prostatic acini with adenocarcinoma. Treatment with LDE-DTX in the PC + LDE + DTX and PC + LDE-DTX + Ex groups significantly reduced the number of acini with adenocarcinoma (Table 2).

**Table 2:** Occurrence of histopathological disorders in experimental animals with PC.

	PC	PC + Ex	PC + LDE-DTX	PC + LDE-DTX + Ex	*p*-value
No changes	35 (630)–5.55%^a^	50 (268)–18.65%^b^	41 (284)–14.43%^ab^	60 (342)–17.54%^ab^	0.0289
LGPIN	99 (630)–15.71%^a^	74 (268)–27.61%^ab^	99 (284)–33.17%^b^	118 (342)–34.50%^b^	0.0479
HGPIN	370 (630)–58.73%^a^	121 (268)–45.46%^ab^	115 (284)–40.49%^b^	140 (342)–40.93%^b^	0.0119
Metaplasia	48 (630)–7.61%^a^	15 (268)–5.59%^a^	33 (284)–11.61%^a^	23 (342)–6.72%^a^	> 0.05
Adenocarcinoma	80 (630)–12.69%^a^	8 (268)–2.98%^b^	1 (284)–0.35%^b^	0 (342)–0%^b^	0.0005

The data are presented as the percentage, the significance of *p* < 0.05 is indicated by *p* value. The results were expressed as absolute value and percentage of occurrences, and the significant differences adopted were *p* < 0.05.

**Figure 3:**
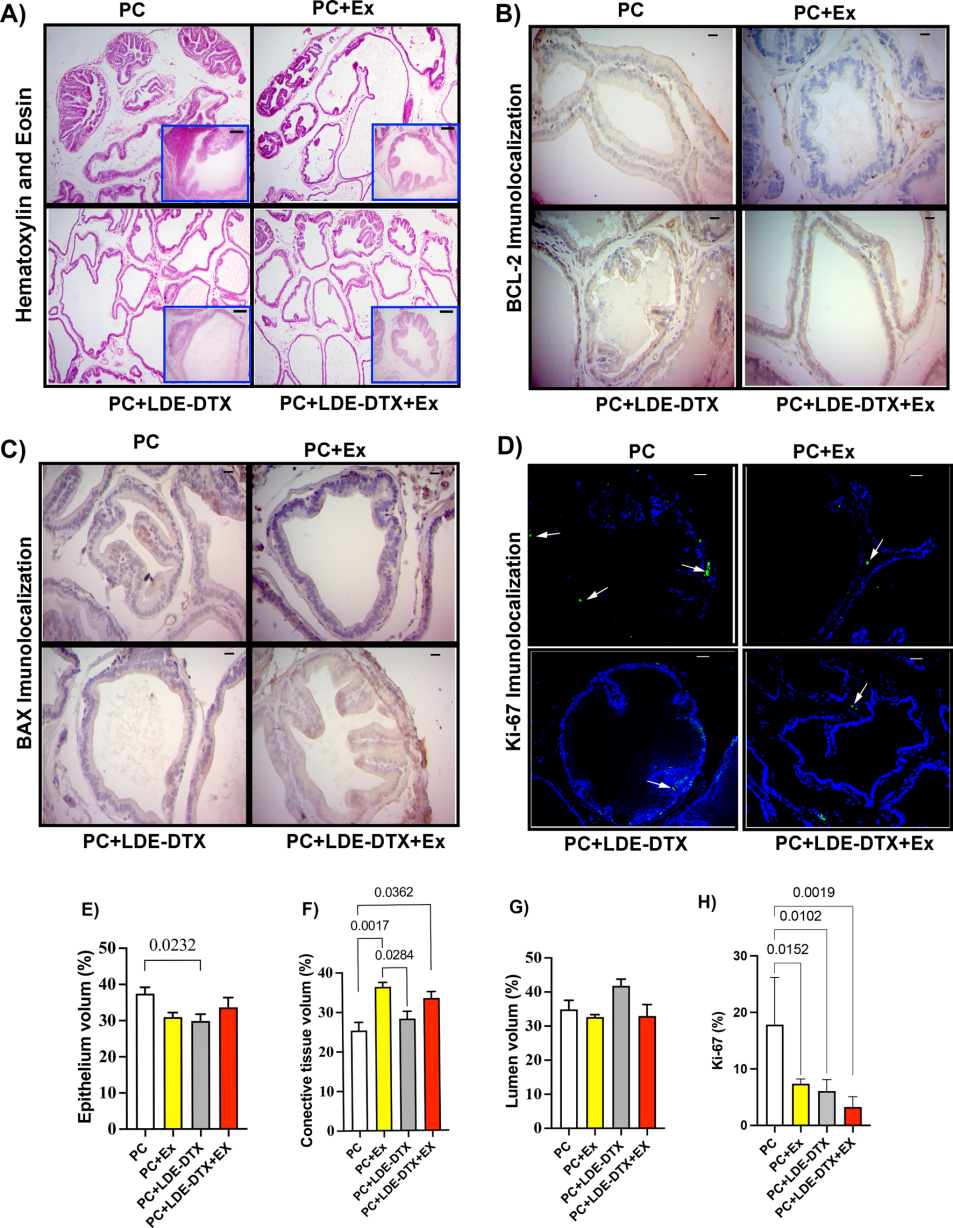
(**A**) Hematoxylin and eosin (H&E) technique developed in the ventral prostate; (**B**) BCL-2 immunohistochemistry protein in the ventral prostate; (**C**) BAX immunohistochemistry protein in the ventral prostate; (**D**) Immunofluorescence of KI-67 protein in ventral prostate; (**E**) Epithelium volume (%) of ventral prostate; (**F**) Connective tissue (%) of prostatic tissue; (**G**) Lumen (%) in prostate samples; (**H**) KI-67 (%); Bar = 20 um, 400×magnification and 100×magnification in image A.

To evaluate prostatic volume with LDE-DTX treatment and physical exercise, we used the Weibel method. Figure 3A,E shows LDE-docetaxel treatment reduced the volume of the epithelium compared to PC group (*p* = 0.0232). In addition, the PC group showed a lower connective tissue percentage compared to PC + Ex, PC + LDE-DTX, and PC + LDE + DTX + EX (Fig. 3A,F). The lumen percentage does not show significant differences (Fig. 3A,G).

### Cell proliferation, cell cycle and apoptosis analysis

To investigate the direct effects of LDE-DTX in combination with physical exercise on cell proliferation, apoptosis, and cell cycle arrest, we employed immunofluorescence and immunohistochemistry staining techniques. Additionally, we quantified the expression levels of relevant proteins involved in these processes using Western blot analysis.

**Figure 4:**
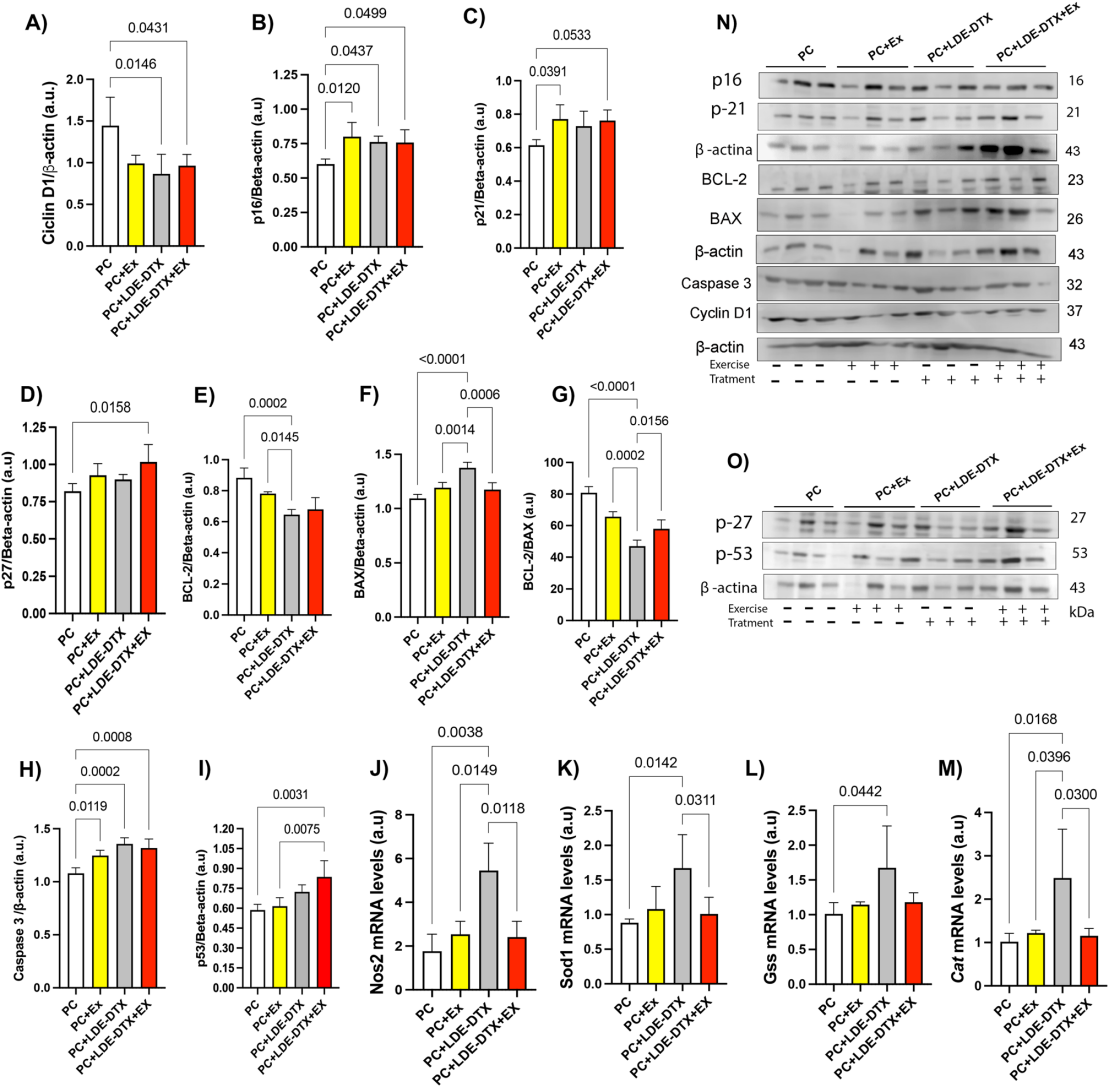
(**A**) Protein levels of Cyclin D-1/β-actin (a.u) in prostatic tissue; (**B**) Protein levels of P-16/β-actin (a.u) in prostatic tissue; (**C**) Protein levels of p-21/β-actin (a.u) in prostatic tissue; (**D**) Protein levels of p-27/β-actin (a.u) in prostatic tissue; (**E**) Protein levels of BCL-2/β-actin (a.u); (**F**) Protein levels of BAX/β-actin (a.u); (**G**) Ratio between BCL-2/BAX (a.u); (**H**) Protein levels of Caspase-3/β-actin (a.u); (**I**) Protein levels of p-53/β-actin (a.u); (**J**) NOS-2 mRNA levels (a.u) obtained by PCR-RT analysis in prostate; (**K**) SOD-1 mRNA levels (a.u) obtained by PCR-RT analysis in frozen prostate; (**L**) GSS mRNA levels (a.u) obtained by PCR-RT analysis in frozen prostate; (**M**) CAT mRNA levels (a.u) obtained by PCR-RT analysis in prostate; au = arbitrary unit; (**N**) Representative protein levels of p-16 and p-21 proteins; BCL-2, BAX, Caspase-3, Cyclin D-1 proteins in Western Blotting; (**O**) Representative Western Blotting of p-27 and p-53 proteins.

Prostate cancer is a histopathologically heterogeneous tumor, and the measurement of Ki-67 antigen serves as a predictor of proliferative activity. Protein expression of the proliferation marker Ki-67 was reduced in the PC + Ex (95% CI 1.87–19.03; *p* = 0.0152) PC + LDE + DTX 95% CI 2.646–20.84; *p* = 0.00102) and PC + LDE-DTX + Ex (95% CI 5.471–23.66; *p* = 0.0019) groups compared to PC group (Fig. 3D,H). PC + LDE-DTX (95% CI 0.1108–1.046; *p* = 0.0146) and PC + LDE-DTX + Ex groups (95% CI 0.01356–0.9489; *p* = 0.0431) showed reduction in cyclin D1 compared to the PC group (Fig. 4A,N). In our study, the groups PC + Ex (95% CI − 0.3556 to − 0.04309; *p* = 0.0120), PC + LDE-DTX (95% CI − 0.3166 to − 0.004085; *p* = 0.0437), and PC + LDE-DTX + Ex (95% CI − 0.3126 to − 8.506e; *p* = 0.0499) demonstrated elevated levels of p16 expression compared to the PC group (Fig. 4B,N). Furthermore, our findings showed greater expression of p21 in PC + Ex (95% CI − 0.2677 to − 0.04495; *p* = 0.0050), PC + LDE-DTX (95% CI − 0.2252 to − 0.002451; *p* = 0.00443), and PC + LDE-DTX + Ex (95% CI − 0.2587 to − 0.03595; *p* = 0.0079) groups in relation to the PC group (Figs 4C,N). We observed greater p27 expression in the PC + LDE-DTX + Ex group (95% CI − 0.3576 to − 0.03577; *p* = 0.0158) compared to the PC group (Fig. 4D,O).

**Figure 5:**
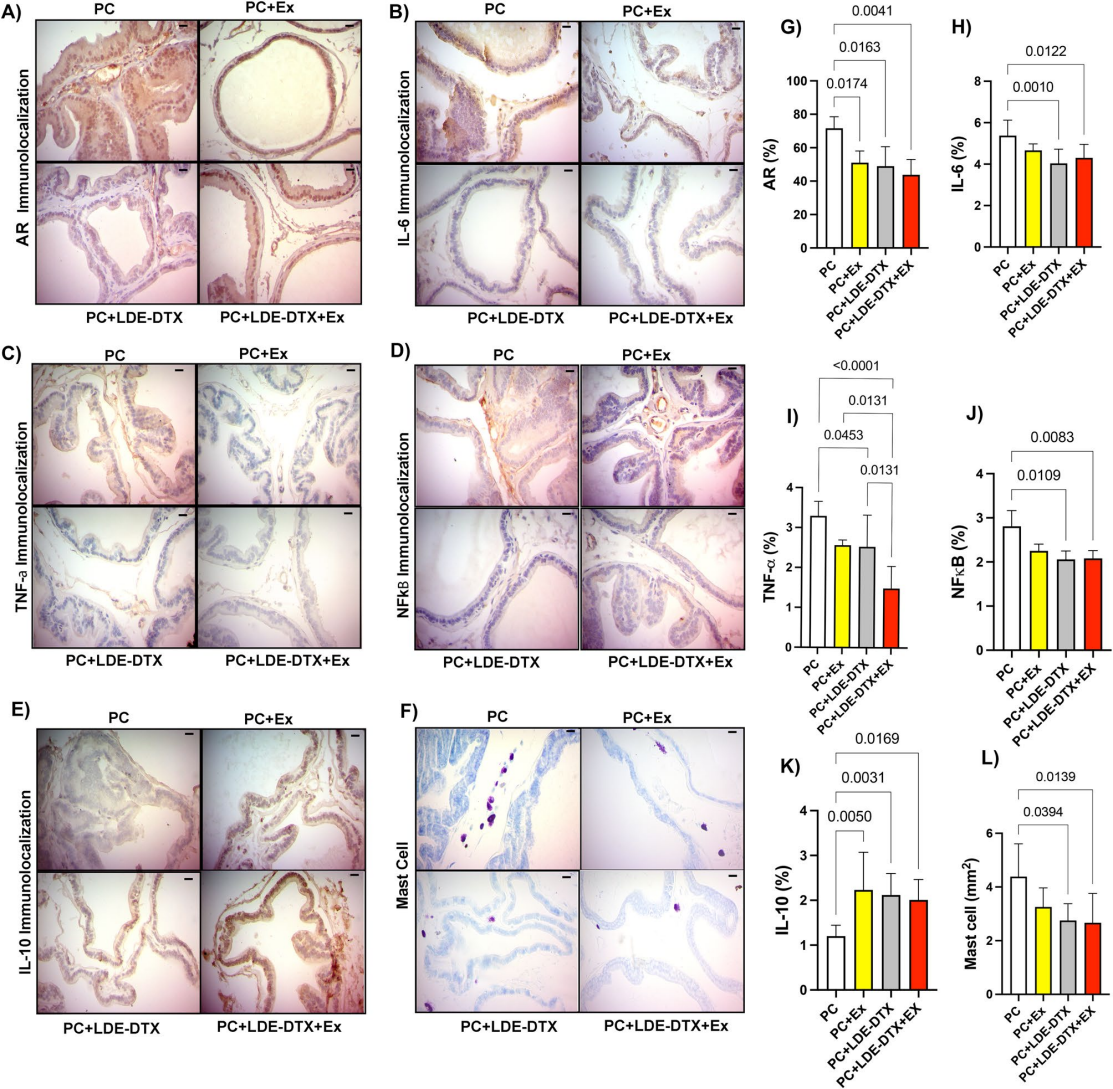
(**A**) AR immunolocalization stain in rats prostate; (**B**) IL-6 immunolocalization labeling in rats prostate; (**C**) TNF-α immunolocalization expression in rats prostate; (**D**) NF-κB immunolocalization in rats prostate; (**E**) IL-10 immunolocalization in rats prostate; (**F**) Mast cells quantity in prostate tissue; (**G**) Percentage of AR labeling in prostate; (**H**) Percentage of IL-6 labeling; (**I**) Percentage of TNF-α labeling; (**J**) Percentage of NF-κB labeling; (**K**) Percentage of IL-10 labeling; (**L**) Mast cells (mm^2^) in ventral prostate. Bar = 20um, and 400×magnification.

The representative western blotting bands of cell death markers in the prostate are showed in Fig. 4N. The PC + LDE-DTX group decreased BCL-2 protein expression compared to PC + Ex (95% CI 0.02616–0.2458; *p* = 0.00145), and PC (95% CI 0.1282–0.3478; *p* = 0.0002) groups (Figs. 3B, 4E,N). Moreover, BAX protein was overexpressed in the PC + LDE-DTX group compared to other groups (Figs. 3C, 4F,N). The ratio of BCL-2/BAX proteins was lower in the PC + LDE-DTX group compared to PC + LDE-DTX + Ex (95% CI − 19.91 to − 2.014; *p* = 0.0156), PC + Ex (95% CI 9.683–27.58; *p* = 0.0002), and PC (95% CI 24.84–42.74; *p* = < 0.0001) groups (Fig. 4G). The protein expression of pro-apoptotic proteins caspase 3 increased expression in PC + EX (*p* = 0.0119) PC + LDE-DTX (*p* = 0.0002) and PC + LDE-DTX + Ex (0.0008) compared to the PC group (Fig. 4H,N). The p53 protein is tumor suppressor was demonstrated a substantial increase in PC + LDE + DTX + Ex treatment group compared to PC (*p* = 0.0031) and PC + Ex (*p* = 0.0075) groups (Fig. 4I,O). Therefore, the amount of positive luminal cells showed that PC + Ex (*p* = 0.0174) PC + LDE-DTX (*p* = 0.163) and PC + LDE-DTX + Ex (*p* = 0.0041) reduced the amount of AR in the prostate compared to the PC group (Fig. 5A,G).

### LDE + DTX increased the expression of Antioxidants

The mRNA levels of *Nos2, Sod1, Cat,* and *Gss* were analyzed in the prostate (Fig. 4). In the PC + LDE + DTX group, there was an increase in *Nos2* mRNA levels *compared to PC* (*p* = 0.0038), PC + Ex (*p* = 0.0149), and PC + LDE + DTX + Ex (*p* = 0.0118; Fig. 4J). The PC + LDE + DTX increased *Sod1* mRNA levels compared to the PC (*p* = 0.0142), and PC + LDE + DTX + Ex (*p* = 0.0311; Fig. 4K). The *Gss* mRNA in PC + LDE + DTX group although was significantly different when compared between PC group (*p* = 0.0442; Fig. 4L). Additionally, *Cat* mRNA levels was increased in the PC + LDE + DTX group when compared to PC group (*p* = 0.0168), PC + Ex (*p* = 0.0396), and PC + LDE + DTX + Ex (*p* = 0.0300; Fig. 4M).

### Inflammation analysis

To investigate the regulation of inflammation within the prostate, we conducted an analysis of mast cell recruitment to sites of inflammation. Notably, the presence of mast cells within the prostate was notably reduced in both the PC + LDE + DTX (*p* = 0.0394) and PC + LDE + DTX + Ex (*p* = 0.0139) groups when compared to the PC group (Fig. 5F, L). Furthermore, to establish a direct link between PC + LDE-DTX treatment and the modulation of the inflammatory state, we conducted immunohistochemistry staining and quantified the levels of key proteins involved in these processes. The animals treated with PC + LDE-DTX (*p* = 0.0010) and PC + LDE + DTX + Ex (*p* = 0.00122) exhibited markedly lower protein expression levels of IL-6 in stark contrast to the PC group (Fig. 5B,H). In relation the TNF-α, the PC + LDE + DTX group was reduced compared to PC + LDE + DTX (*p* = 0.0131), and PC + Ex (*p* = 0.0131; Fig. 5C,I). The PC group increased TNF-α in comparation to PC + LDE + DTX (*p* = 0.0453), and PC + LDE + DTX + Ex (*p* = 0.0001; Fig. 5C,I). NF-κB markers, in stark contrast to the PC in related to PC + LDE + DTX (*p* = 0.0109), and PC + LDE + DTX + Ex groups (*p* = 0.0083; Fig. 5D,J).

To ascertain the anti-inflammatory potential of the treatment on the prostate, we assessed interleukin-10 (IL-10) expression. IL-10, a cytokine crucial for maintaining immune response equilibrium, demonstrated enhanced expression levels in the prostate tissues of the PC + LDE-DTX (*p* = 0.0031), PC + LDE + DTX + Ex (*p* = 0.0169), and PC + Ex (*p* = 0.0050) groups in comparison to the PC group (Fig. 5E,K).

## Discussion

To the best of our knowledge, this study represents the pioneering documentation of in vivo effects of a docetaxel-loaded nanoparticle in prostate tumors, in association with physical exercise. Within this study, the administration of LDE-DTX demonstrated remarkable success in diminishing tumor volume in this rat-induced model of prostate cancer, all without discernible toxic effects. Rats afflicted with prostate cancer exhibited a substantial elevation in the incidence of invasive growth metastatic adenocarcinomas within the prostate^17^. Presumably, this model mirrors the aggressive tumor progression accompanied by escalated proliferation. Our findings are in concurrence with prior reports showcasing the impact on tumor volume attributed to LDE nanoparticle conjugation with chemotherapy agents^5,18^. We provide compelling evidence of LDE-docetaxel efficacy in reducing prostate mass, adenocarcinoma, and high-grade neoplasms. Another key finding of this study was the fact that aerobic physical exercise simultaneously to LDE-DTX treatment had the capacity of increasing the reduction of the tumor volume produced by LDE-DTX. Furthermore, in conjunction with physical exercise, LDE-docetaxel demonstrated an augmented internalization of LDE [14C]-cholesteryl ester within the prostate, contributing to further tumor volume reduction. The outcomes achieved through the potent antiproliferative agent DTX, in conjunction with LDE, hold the potential to illuminate novel pathways in the pursuit of overcoming prostate cancer, while ensuring treatment efficacy over extended periods.

As shown by our results, elevated levels of circulating cholesterol and lipids were positively linked to the development of PC. LDE-docetaxel treatment indicated a potential reduction in lipid levels in the blood regardless of whether they underwent physical exercise. Cancer cells show metabolic reprogramming, mainly affecting energy metabolism^19^. Nevertheless, neoplastic cells in prostate exhibit alterations in the metabolic pathways involving fatty acids, triglycerides, and cholesterol^20^. Furthermore, LDE-DTX treatment demonstrates elevated levels of HDL and decreased values of triglycerides and glucose. Data from a recent meta-analysis utilizing epidemiological studies found that high blood concentrations of cholesterol and triglycerides, coupled with low concentrations of HDL-C, are associated with an increased risk of overall cancer^21^. Experimental studies have shown that HDL-C can protect against tumor development through various mechanisms, such as influencing signaling pathways by modulating cholesterol content in cell membranes^22^. Experimental studies have also revealed that HDL-C possesses antioxidant and anti-inflammatory properties and plays a role in inhibiting the LDL oxidation cascade^23^. Thus, the protective effects of LDE-docetaxel through HDL may mitigate prostate cancer progression. Similarly, we have observed the metabolic effect of physical exercise, resulting in increased energy metabolism due to heightened muscular activity. Therefore, low levels of blood lipids could serve as an indirect measure of response to LDE-docetaxel treatment combined with exercise, reflecting a decrease in this metabolic activity with slowed tumor growth. These findings potentially hold translational significance, as they indicate that reducing cholesterol may be beneficial in lowering the risk of prostate cancer associated with LDE-DTX treatment.

We found a significant increase in the values related to the 646 cm^−1^ band and the 1003 cm^−1^ band by Raman spectroscopy related to tyrosine and phenylalanine in the groups treated with LDE-docetaxel with or without physical exercise. Metabolic biomarkers can be used for PC diagnosis. These compounds include amino acids such as phenylalanine and tyrosine, that were identified as diagnostic indicators for PC in body fluids^24,25^. Phenylalanine is the main metabolic pathway that yields the amino acid tyrosine, which is involved in the production of melanin and thyroxine. Tyrosine as a cancer marker has been studied in different types of cancer. Miyagi et al.^26^ showed a significant decrease in tyrosine levels in plasma samples from patients with gastric cancer compared to controls, and the same pattern was also apparent between early and advanced stage gastric cancer. In the same way, Zhang et al.^27^ showed decreased serum tyrosine levels in patients with esophageal cancer compared to healthy controls. Thus, it is possible to verify that the treatment with LDE-docetaxel increased the concentration of biomarkers indicating alteration in the metabolism of phenylalanine related to the reduction of the PC.

Furthermore, we investigated the effect between LDE-docetaxel and physical exercise in increasing the activation of p53 pathways, which has been shown to play a central role in inducing apoptosis and interrupting DNA replication, thus mediating cell cycle arrest. Previous studies have indicated the crosstalk between p53 and AR signaling, where the overexpression of p53 inhibits expression of androgen-dependent genes^28^. Activation of p53 and subsequent transcription of several p53 target genes, including p21, induces long-term cell cycle arrest or apoptosis. Treatment with LDE-docetaxel associated with exercise considerably increased p53 expression and reduced AR levels in PC. After activation of p53 pathways, pro-apoptotic proteins (BAX and caspase 3) were up-regulated and anti-apoptotic proteins (BCL-2) were down-regulated in groups treated with LDE-DTX and LDE-DTX + Ex. Caspase expression and activation represent an important cellular marker of apoptosis^29^ since the loss of apoptotic control in association with cell proliferation is responsible for the onset and progression of PC^30^. Altered caspase expression may represent an additional component related to cell death promoted by LDE-docetaxel treatment. Taken together, the data presented in this work indicates that LDE-docetaxel associated or not with physical exercise induces caspase-3-mediated apoptosis with changes in the BCL-2/BAX ratio. Therefore, our findings provide a link between LDE-docetaxel treatment and histopathological reduction of prostatic adenocarcinoma. By restricting these proteins, the proliferation, angiogenesis and anti-apoptosis abilities of neoplastic cells were all reduced.

**Figure 6:**
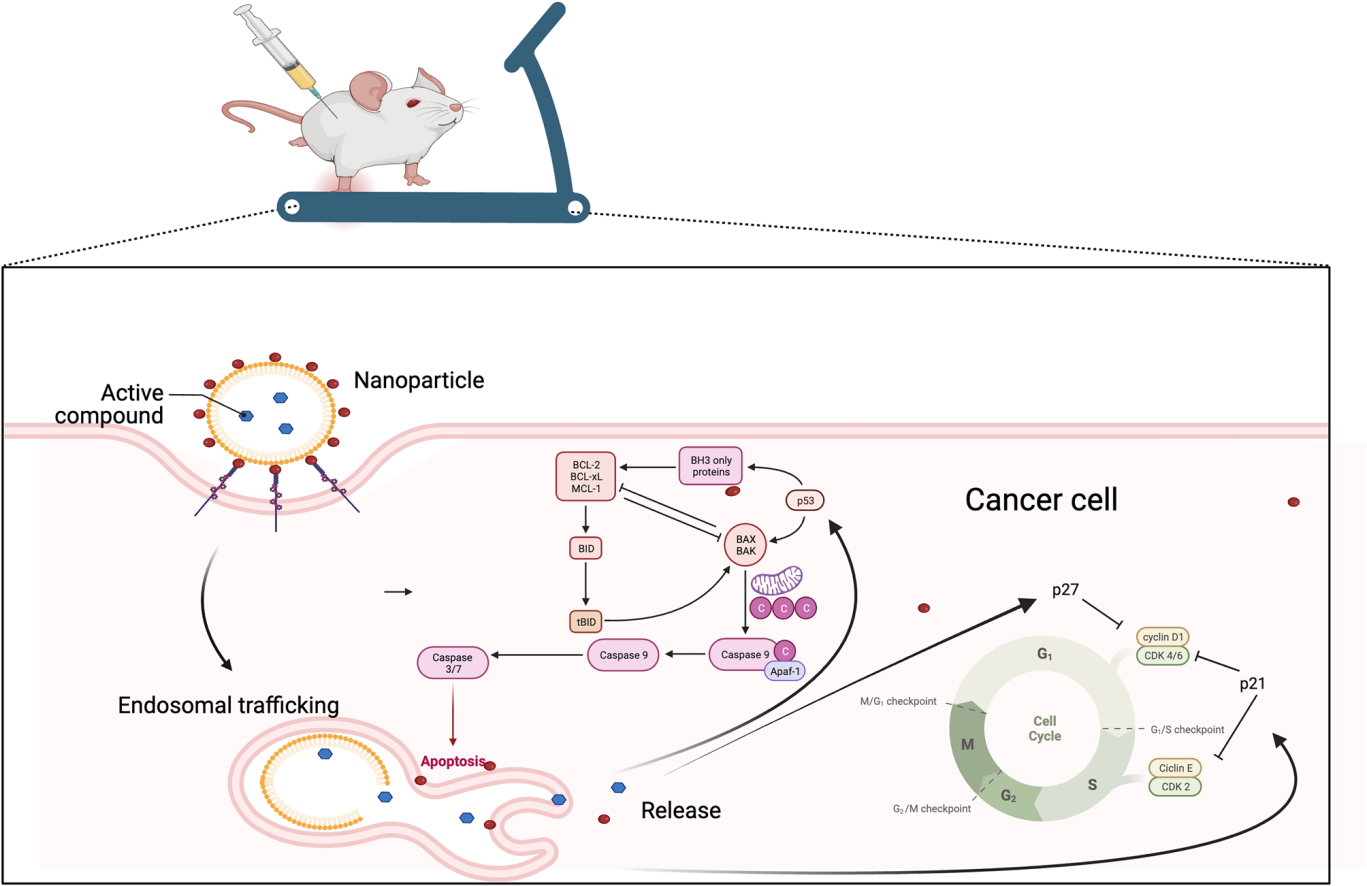
Graphical abstract that illustrates the treatment with LDE-docetaxel led to a decrease in cell proliferation with an increase in cellular apoptosis. This effect was amplified when combined with aerobic physical exercise. An increase in the cell cycle inhibitory proteins p-21 and p-27 can be observed, indicating an interruption in the G1/S and G2/M cell cycle transitions, suggesting a blockade in cell cycle progression at the G0/G1 phases. Elevated p-53 due to the treatment alters the apoptosis pathway by increasing the expression of BAX and caspase 3 and decreasing BCL-2. This image was created using Bio render software, https://www.biorender.com/.

For better understand the combined effect of LDE-docetaxel in an induced PC model, we analyzed cell cycle proteins after treatment. First, we observed a reduction in cell proliferation (Ki-67 expression) after treatment with LDE-docetaxel, reduced when associated with physical exercise (Fig. 3). To understand the effect of treatment on the cell cycle we analyzed cyclin D1 and CDK inhibitors p21 and p27, and we verified an increase in p21 and p27 protein in the treated groups. The p21 is a potent cyclin-dependent inhibitor that regulates G1/S phase progression and is tightly regulated by the tumor suppressor p53^31^. CDK 4 and CDK 6 are cyclin D1-regulated cell cycle proteins that are critical for cell cycle progression through G1 phase^32^. In our study we found the negative regulation of cyclin D1 and the positive regulation in the expression of p21 and p27 indicating a clear arrest of the G0/G1 cell cycle in the groups with LDE-docetaxel treatment and physical exercise. Considering that p21 and p27proteins inhibit mainly G1-S cyclin/Cdk complexes, we speculate that LDE-docetaxel may be affecting the cell cycle of PC, with modulation in proliferation arrest through disruption of the G1/S and G2/M transitions. However, the enhanced effect observed when the treatment with LDE-DTX was combined with aerobic physical exercise resulted in a significantly higher inhibition of cell cycle progression when analyzing the p53 protein, compared to treatment with LDE-docetaxel alone, which is innovative (Fig. 6).

In this study, LDE-DTX increased *Nos2* and antioxidant enzymes. Within the tumor microenvironment, sustained supraphysiological concentrations of ROS overwhelm coping mechanisms, leading to a state of oxidative stress^33^. ROS generation is inherently countered by increased antioxidant capacity, where LDE + DTX nanoemulsion significantly elevated mRNA levels of *Cat*, *Sod1*, and *Gss*. The protocol used to induce tumors using DMBA is based on the premise of increasing harmful biological events, promoting changes in ROS^34^. ROS generated during treatment with LDE + DTX act as important subcellular messengers, stimulating various kinases involved in gene expression and cellular adaptation^35^.

Indeed, it is noteworthy that the action of LDE-DTX exhibited favorable outcomes across all molecular aspects of anti-carcinogenesis examined in this study. The expression of TNF-α and IL-6 proteins ranks among the most well-established pro-inflammatory cytokines, which were inhibited through LDE-DTX treatment in PC. Inflammation is closely intertwined with the development and progression of prostate cancer^36^. Treatment with LDE-DTX resulted in diminished mast cell quantities, coupled with reduced levels of IL-6, TNF-α, and NF-κB in PC. In this study, LDE-DTX treatment is associated with reduced adenocarcinoma, accompanied by decreased proliferation and increased apoptosis, as well as diminished inflammation, particularly when coupled with physical exercise. On the other hand, we discovered the anti-inflammatory effect of LDE-DTX, characterized by elevated levels of IL-10 in the prostate. IL-10 is recognized for its regulation of the immune system, suppressing exacerbation of the adaptive immune system response, and preventing tissue damage through immune response^37^. Administration of IL-10 to lymphoma-bearing mice significantly curtailed tumor growth, with a noteworthy rise in memory CD8 + T cells in comparison to non-IL-10-injected mice, suggesting IL-10’s to enhance anti-tumor effects, immunity, and conceivably play a role in cancer recurrence prevention^38^. Hence, LDE-DTX and LDE-DTX + EX exhibit an immunosuppressive effect within the tumor microenvironment, modulated by both inflammatory and anti-inflammatory cytokines.

This article presents compelling evidence regarding the application of a chemotherapeutic drug-carrying nanoparticle, LDE, which exhibits a robust affinity for cellular LDL receptors. Our findings unequivocally demonstrate that LDE uptake is significantly augmented following physical exercise, subsequently exerting a profound influence on the metabolism of neoplastic cells (refer to Fig. 6). This research carries substantial significance in delineating an optimal treatment strategy for prostate cancer, as the synergistic interplay between physical exercise and small lipid particles, such as LDE with docetaxel, holds the potential to induce cell apoptosis through the p53 protein pathway in relation the PC and PC + Ex groups. Furthermore, the regulation of lipids and glucose utilization induced by physical exercise at a systemic level led to notable alterations in amino acid expression, thereby underscoring the extensive metabolic implications of the LDE-DTX and physical exercise interaction in PC development. The data elucidated in this study not only emphasizes the promising potential of LDE-DTX in prostate cancer treatment but also underscore its efficacy, but our findings unveil substantial reductions in tumor volume, diminished proliferative capacity, cell cycle arrest, and mitigation of inflammation. These outcomes underscore the significant therapeutic potential of LDE-DTX, hinting at its efficacy in restraining the progression of prostate cancer. Furthermore, the incorporation of physical exercise into the treatment regimen has the potential to amplify these effects, thereby unveiling avenues for complementary strategies that could further enhance patient outcomes. However, in conjunction with physical exercise and as a standalone therapeutic approach.

## Conclusion

In conclusion the results of this study support the safety and effectiveness of LDE-DTX treatment to achieve prostate cancer regression in an experimental model of the disease. Increase in the anti-cancer action of LDE-DTX by simultaneous aerobic physical exercise was also shown. Those findings pave the way for future clinical studies aiming to introduce LDE-DTX in the oncological practice, with exercise training as an advisable supplementary measure to this drug-targeting therapy.

## Materials and methods

### Animals and treatments

Sixty male Sprague–Dawley rats (40-day-old, weighing ± 120 g), were obtained from the Multidisciplinary Center for Biological Investigation (CEMIB) at the University of Campinas (UNICAMP). Animals were housed 3–4 in polypropylene cages with laboratory-grade pine shavings as bedding and kept in a climatized room under controlled temperature at 22 ± 3 °C with a 12/12 h light/dark cycle, lights switched on at 7:00 a.m. During the procedures, standard rodent food (Nuvilab, Colombo, Paraná, Brazil) and filtered tap water were provided ad libitum.

At 50 days of age, the animals were randomized in 6 groups and received a single dose of the carcinogen DMBA (Sigma Aldrich San Luis, Missouri, EUA®) intraperitoneal injection (i.p) of 65 mg/kg of body weight dissolved in sesame oil). During the period of tumor development, ultrasound was performed in the region to verify the development of the tumor mass (Supp 1). After the PC was developed (140-days-old) the animals designated to receive were administered i.p. 6 doses of LDE-docetaxel at 2 mg/kg/week. The animals assigned to perform aerobic physical training for 8 weeks started the treatment with LDE-docetaxel after the third week of the beginning of the physical training protocol. LDE-docetaxel dosages for the group submitted to physical exercise were performed 12 h after the last physical training session. The treatments occurred simultaneously (Fig. 1P). The animals were allocated top 6 experimental groups (n = 10): PC: rats with prostate cancer; PC + LDE*: rats with PC received single LDE dose; PC + LDE + Ex*: animals with PC received LDE single dose and exposed to aerobic physical training; PC + Ex: animals with PC was performed aerobic physical training for 8 weeks; PC + LDE-DTX: animals with PC received 6 LDE-docetaxel doses; PC + LDE-DTX + EX: animals with PC received 6 LDE-docetaxel doses and performed aerobic physical training for 8 weeks.

### Ethics statement

All animal procedures were conducted in accordance with the ethical principles in animal research adopted by the Brazilian College of Animal Experimentation (COBEA) and the study protocol was approved by the Ethics Committee on Animal Use (CEUA) was approved by the Animal Use Ethics Council (CEUA) of the Faculty of Science and Technology (FCT/UNESP) under protocol number 02/2020. This study has been reported in accordance with ARRIVE guidelines. All reports were performed in accordance with relevant guidelines and regulations and legislations.

### Derivatization of docetaxel and preparation of LDE-DTX

To improve the association rate of DTX to LDE, the drug was derivatized to DTX oleate to enhance the lipophilicity, as described previously^5^. LDE was prepared from a mixture of 64% phosphatidylcholine, 33% cholesterol oleate, 1% non-esterified cholesterol and 2% triglycerides^5^. The aqueous phase consisted of 100 mg polysorbate 80 (Merck, Hohenbrum, Germany) and Tris–HCl buffer pH 8.05 was kept at room temperature. Emulsification of all lipids and the aqueous phase was obtained by high-pressure homogenization using an Emulsiflex C5 homogenizer (Avestin, Ottawa, Canada) for 30–40 min. To prepare LDE-DTX, DTX was added to the lipid mixture at a drug:lipid ratio of 1:10 that was then centrifuged at 1800×*g* for 15 min to separate the unbound DTX that precipitates upon centrifugation. The particle size of both preparations was 60 nm, as measured by dynamic light scattering method at a 90° angle, using the ZetaSizer Nano ZS90 equipment (Malvern Instruments, Malvern, UK). The efficiency of the association of DTX to LDE was measured by HPLC. The nanoparticles were sterilized by passing through a 0.22 μm pore polycarbonate filter (EMD Millipore, Billerica, MA, USA) and kept at 4 °C until it was used.

### Incremental load test

The trained groups were submitted to the incremental load test to identify the maximum power of the animals, with this individualized data we stipulated an overload of 60% of the maximum speed, considered of moderate intensity for the performance of the aerobic physical exercise. The test was carried out at 141 days of age in the adaptation period of the animals on the treadmill (beginning), the second test after the 4th week of physical training (during), and the third test in the 8th week of physical exercise (final). The animals were adapted to aerobic physical exercise on a treadmill (Inbramed®, Presidente Prudente, São Paulo, Brazil). The incremental load test started with a speed of 6 m/min, with 0% inclination and increments of 3 m/min every 3 min performed until voluntary exhaustion of the rats. which occurred when the rats touched 5 times at the end of the treadmill in the period of 1 min. The maximum power (Pmax), defined as the animal’s exhaustion speed (m/min), was used to prescribe the physical training intensities^39^.

### Treadmill protocol

At 141 days of age, the rats started the adaptation protocol to aerobic physical exercise, and, at 147 days of age, the animals started aerobic physical training. The chronic aerobic physical exercise protocol was based on Ferreira^39^, consisting of 8 weeks, being each experimental week consisted of 5 consecutive days of training and 2 days of rest, with an overload of 60% of maximum speed. To readjust the load, incremental tests were carried out every 4 weeks.

### Nutritional data

To monitor the biometric data, we assessed changes in food intake and body composition in the rats. Weekly measurements of body weight were taken, calculating the weekly weight change (Δ = final weight − initial weight). To determine the rat’s total energy consumption, we used the food intake value and the caloric value of the rodent feed (3 kcal/g). The total energy consumption (TEI) was calculated as the average food consumption per day (in grams) multiplied by 3 (kcal/day = average food consumption per day [g] × 3). Additionally, we calculated the feed efficiency (EF) as the mean body weight gain divided by the total mean TEI (g/kcal = mean body weight gain/total mean TEI)^40^. We determined the amount of body fat by the sum of epididymal, retroperitoneal and mesenteric fat, data expressed in grams.

### Biochemistry analysis

Blood samples were collected from the rats, then centrifuged and stored for subsequent biochemical assays. We measured concentrations of glucose, total cholesterol, HDL-cholesterol, and triglycerides using an enzymatic-colorimetric method, specifically the Trinder method. All procedures were performed in accordance with the established protocols provided by Gold Analisa Diagnóstica®, located in Belo Horizonte—MG, Brazil.

### LDE uptake

To assess how LDE can reduce prostate cancer, we used an assay with radioactively labeled LDE and verified whether physical exercise would increase the percentage of uptake of the molecule. To perform this analysis, we used two groups: PC + LDE* (PC submitted to doses of LDE); PC + LDE + Ex* (PC and submitted to aerobic physical training and LDE doses for uptake analysis); the animals in the PC and PC + Exercise groups received an intraperitoneal injection of LDE labeled with OC-14C and after 24 h the tissues were collected and prepared for cell capture by the FOLCH method^41^. The group with physical exercise received the LDE dose after 12 h of the last session where the lipid clearance is higher.

### Prostate cancer histopathology

At 189 days of age the animals were submitted to euthanasia per decapitation and the ventral prostate was removed, weighed, and embedded in paraplastic and the 5 μm sections were cut. These sections were then stained with hematoxylin and eosin (H&E), the ventral prostate was evaluated, and the histological fields were photographed, resulting in 40–60 fields per animal analyzed. The slides were photographed in an optical light microscope, model AxioCam ECR5s Zeiss®. To perform the histopathological analysis, we verify the LGPIN, HGPIN, metaplasia, and adenocarcinoma focus performed based in Shappell analysis^42^. The H&E staining was used for stereology analysis using the Weibel method^43^, to this was used 50 slides per group, used a retinaculum of 136 points.

### Immunohistochemistry

The immunohistochemistry technique was performed as previously described by our research group^14^. The sections were subjected to solutions of ImmPRESS Polymer kit (MP-7800) from Vector Laboratories®, for peroxidase and protein blocks. In the next step, the sections were subjected to a reaction with specific primary antibodies diluted 1:100, BAX (B-9, sc-7480) mouse monoclonal IgG, BCL-2 (C-2, sc-7382), Nuclear factor kappa B, NF-κB (p-65, A, sc-109), interleukin 6, IL-6 (E-4, sc-28343), tumor necrosis factor alpha, TNF-α (52B83, sc-257), androgen receptor, AR (441, sc-7305), and interleukin 10, IL-10 (NYRM, sc-73309), mouse monoclonal IgG, and incubated in a humid chamber overnight. The samples were incubated with a secondary monoclonal antibody, m-IgGk HRP (sc-516102), at room temperature, developed with diaminobenzidine (DAB), stained with Harris hematoxylin and with a Zeiss Axiophoto photomicroscope (Zeiss, Munich, Germany). The intensity of BAX and BCL-2 antigens immunoreactivity were examined in 10 fields per animal using Image-J software version 1.50i (NIH, Bethesda, MD, USA), and % of tissue marking was quantified for each image, and immunopositivity cells were used for area percentage.

### Immunofluorescence

The prostate sections were subjected to reaction with specific primary antibody KI-67 1:50 (ab-16667) and incubated in a humid chamber overnight. After washing, all sections were incubated for two hours at room temperature with FITC 1:100 (goat anti-mouse, Invitrogen NOVEX), and the DAPI 1:50 was applied for thirty minutes (DAPI 306, Vector Laboratories®). The sections were mounted with Vectashield (H-1000, CA94010 Burlingame, Vector Laboratories) and examined using an inverted confocal microscope. The intensity of immunoreactivity of KI-67, antigens were examined in 10 fields per animal using Image-J software version 1.50i (National Institutes of Health, Bethesda, MD, USA), and the number of cells marked was quantified in 1000 cells and used for percentage of positive cells calculation.

### Western blotting

Ventral prostate samples from each experimental group were homogenized with RIPA buffer and 10% protease inhibitor buffer, Bradford method to quantify the protein, subjected to SDS—polyacrylamide gel (SDS—PAGE) electrophoresis and then transferred from the gel to a nitrocellulose membrane. Subsequently incubated with Santa Cruz Biotechnology® primary antibodies of BAX 1:1000 (B-9, sc-7480), BCL-2 1:1000 (C-2, sc-7382), P-16 1:1000 (JC8, sc-56330), P-21 1:1000 (187, sc-817), P-27 1:1000 (F8, sc-1641), P-53 1:1000 (DO-1, sc-126). Then, the samples were incubated with m-IgG secondary antibody (BP-HRP, sc-516102, HRP conjugated) and revealed with Cytiva Amersham™ ECL™ Prime with C-Digit Digital Western Blot Imaging. The band expression (animals/group) was quantified by Image Studio software, the values of each protein then were normalized by the values of the respective β-actin 1:500 (sc-47778).

### Reverse transcription-quantitative polymerase chain reaction (RT-q-PCR)

The prostate samples were immersed in Trizol and crushed in a tissue homogenizer, then stored at -80ºC. The concentration of total RNA was measured by spectrophotometry and subjected to DNAse treatment according to the instructions provided with DNAse I-Amplification Grade. Reverse transcription followed the high-capacity protocol. The expression levels of genes encoding nitric oxide synthase 2 (*Nos2*), catalase (*Cat*), glutathione synthetase (*Gss*), superoxide dismutase (*Sod1*) was assessed via real-time PCR. To normalize the relative expression of these target genes, the mean expression values of the GAPDH gene were employed^44^. Real-time PCR amplification was conducted using an Applied Biosystems 7500 Real-Time PCR Systems thermocycler. Calibration curves for each gene were established using serial dilutions of a cDNA pool synthesized from 20 µg of prostate mRNA.

### Raman spectra

The potential applications of Raman spectroscopy in biology are extensive. This technique allows for molecular-level analysis, enabling the study of functional groups, bond types, molecular conformations, and direct biochemical composition. In this study, blood serum was used for the determination of Raman spectra. The Raman spectra were acquired using a handheld Raman instrument (Bruker, model BRAVO) equipped with a Duo LaserTM system operating at wavelengths of 785 and 852 nm, along with SSETM for fluorescence reduction. To obtain the Raman spectra of the serum, 2 mL of the sample was added to a cuvette in the handheld Raman holder. The integral values of the band areas in the 417, 454, 505, 646, 1003, 1444, and 1653 cm-1 region of the Raman spectra were calculated using the GRAMS/AI™ spectroscopy software.

### Multidimensional projection

The Raman spectra were subjected to analysis using a multidimensional projection technique, which allows data from a higher-dimensional space to be projected into a two-dimensional (2D) space while preserving similarity relationships. Detailed information about this projection technique can be found in the works of Tejada^45^ and Minghim^46^. In general, the 2D plot is created by projecting them into the plane with graphical markers represented by Y = {y_1_,y_2_,…,y_n_}, with the positions on the 2D plot being determined in an optimization procedure using an injective function f: X → Y that minimizes |δ(x_i_, x_j_) − d(f(x_i_),f(y_j_))| ≈ 0, ∀x_i_, x_j_ ∈ X^45^, where d(y_i_,y_j_) is the distance function on the projected plane. The flexibility of this optimization approach arises from the availability of several cost (or error) functions used for placing the graphical markers on the 2D plot. Here we used the so-called Interactive Document Map (IDMAP)^46,47^, whose function is defined as follows:

$$S_{IDMAP} = \frac{{\delta (x_{i} ,x_{j} ) - \delta_{\min } }}{{\delta_{\max } - \delta_{\min } }} - d(y_{i} ,y_{j} )$$

where ***δ*** and ***d***, are the distance functions defined above and *δ*_min_ and *δ*_max_ are the minimum and maximum distances between samples^48^.

### Statistical analysis

GraphPad Prism v.9 was used for statistical analysis of functional data and for graphing. Shapiro–Wilk test to verify the normal distribution. We, therefore, used analysis of variance of bidirectional estimated marginal means (ANOVA) complemented by Tukey’s multiple comparisons test to confirm statistically significant differences for normally distributed results (*p* < 0.05) and repeated measures. For non-parametric tests, we used Kruskal–Wallis with Dunnet’s post-test (*p* < 0.05). The p-value in the graph indicates the mean ± standard deviation.

### Supplementary Information

Supplementary Figure S1.

## Data Availability

The data used during this study are available from the corresponding author upon reasonable request.
