# Supplementary material for: Integrated aerobic exercise with LDE-docetaxel treatment: a novel approach to combat prostate cancer progression
Source: Sci Rep. 2024 Apr 26;14:9626. doi: 10.1038/s41598-024-60138-y (PMC11053171; doi:10.1038/s41598-024-60138-y)
Supplement: Supplementary file 1 — Supplementary Figure S1. [file 41598_2024_60138_MOESM1_ESM.docx]

**
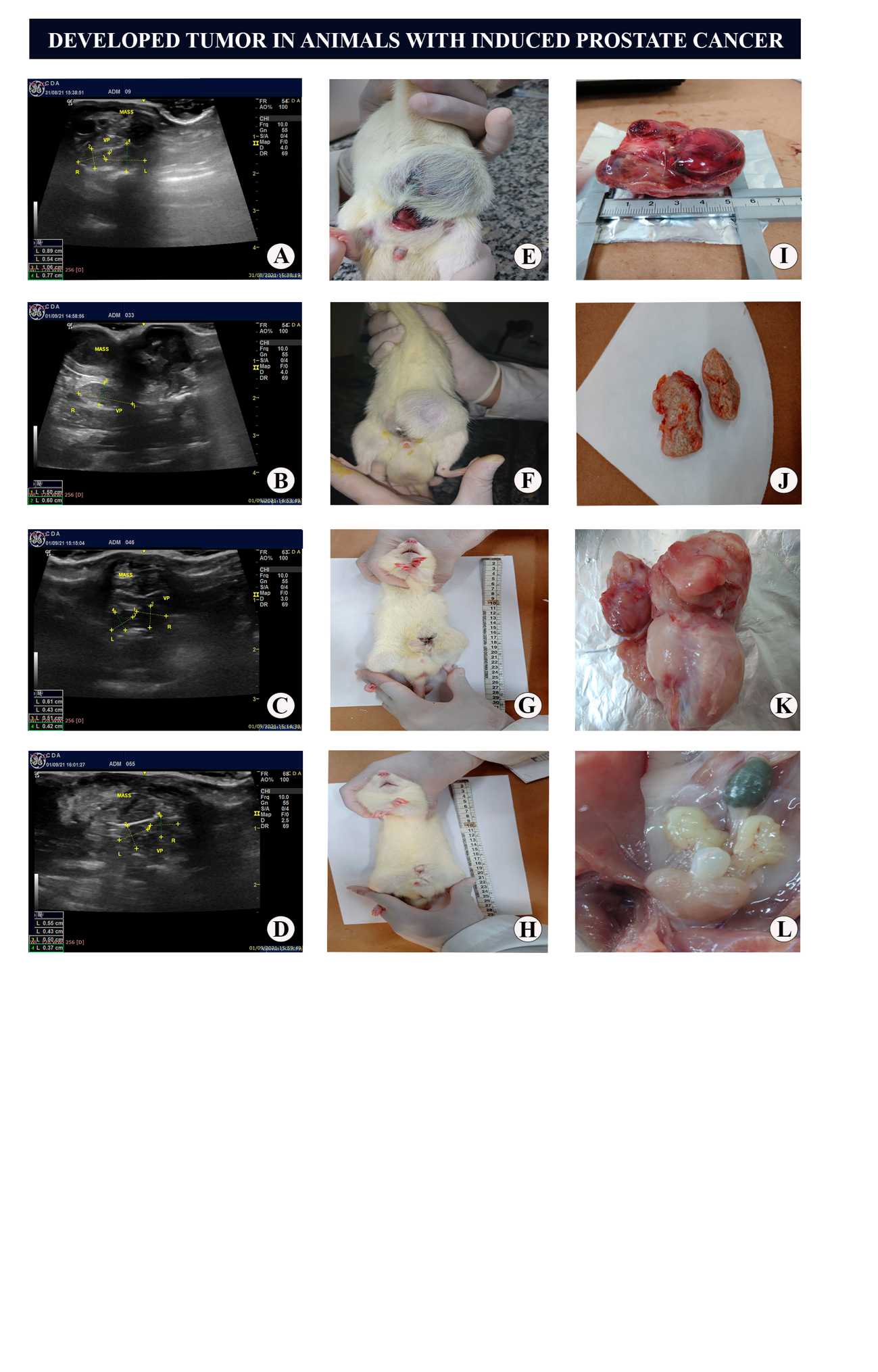
**

**Figure S1.** Supplementary Figure (S1): **(A-D)** Images referent to ultrasound analyzes in treated and untreated animals (PC, PC+EX, PC-LDE-DTX, and PC-LDE-DTX+EX), along the experimental protocol; VP = ventral prostate; R = right side of animal body; L = left side of animal body; Mass = tumor size and diameter; **(E-H)** Illustrations of inside developed tumors in Sprague-Dawley rats at final moment of protocol; **(I-L)** Representations of developed tumors in Sprague-Dawley rats in cadaveric moment (post euthanasia).
